# Association between the use of herbal medicines during pregnancy and adverse fetal outcomes among mothers in eastern Ethiopia, 2023

**DOI:** 10.1016/j.heliyon.2024.e40941

**Published:** 2024-12-06

**Authors:** Tamirat Getachew, Abera Kenay Tura, Merga Dheresa, Shamble Niguse, Bikila Balis, Lemma Demissie Regassa, Addis Eyeberu

**Affiliations:** a School of Nursing and Midwifery, College of Health and Medical Sciences, Haramaya University, Harar, Ethiopia; b School of Pharmacy, College of Health and Medical Sciences, Haramaya University, Harar, Ethiopia; c School of Public Health, College of Health and Medical Sciences, Haramaya University, Harar, Ethiopia

**Keywords:** Integrative medicine, Traditional african medicine, Herbal medicines, Pregnant women, Adverse fetal outcomes, Ethiopia

## Abstract

**Background:**

Nowadays, pregnant women around the world use herbal remedies extensively. Evidence illustrated that the association between the use of herbal medicines and unfavorable fetal outcomes is not well established. Furthermore, much of the existing research is conducted within medical facilities, which may result in excluding mothers who do not receive antenatal follow-up care. Therefore, this study aimed to explore the association between the use of herbal medicine and adverse fetal outcomes in eastern Ethiopia.

**Methods:**

A community-based cross-sectional study was conducted among 882 randomly selected mothers from February 1, 2023 to November 1, 2023. Data were collected through face-to-face interviews using structured questionnaires adapted from previous literature. The dataset was analyzed using Stata version 18, and a modified Poisson regression model was applied. Adjusted prevalence ratios (APR) with 95 % confidence intervals were estimated.

**Results:**

The prevalence of herbal medicines use among pregnant women in eastern Ethiopia was 43.2 % (95 % CI: 39.9, 46.5). The most commonly used herbal medicines were *Ocimum lamiifolium* 109 (29.1 %), *pumpkin fruit* 68 (18.2 %) and *Rutachalepensis* 61(16.3 %). Factors such as rural residency [APR = 1.27, 95%CI (1.01,1.59)], having obstetric complications in recent pregnancy [APR = 1.42, 95 % CI: (1.14,1.78)], and experiencing adverse fetal outcomes [APR = 1.59, 95%CI: (1.05,2.2)] were significantly associated with the use of herbal medicine.

**Conclusions:**

More than two out of five mothers used herbal remedies during their recent pregnancy. The study identified a significant association between the use of herbal medicines and various sociodemographic and obstetric factors. Consequently, it is essential to establish the safety and effectiveness of herbal medications before their use during pregnancy. To address the diverse health needs of pregnant women and mitigate the risks associated with the indiscriminate use of herbal remedies, a collaborative strategy should be developed involving herbalists, communities with strong beliefs in herbal medicine, and healthcare professionals.

## 1 Introduction

Globally, pregnant women are increasingly turning to herbal drugs. Most pregnant women use herbal medicine to treat diseases and improve their health before, during, and after giving birth [1]. Numerous studies have indicated that the use of herbal remedies during pregnancy is quite common. Studies from the UK (56 %) [2], Italy (48 %) [3], and Norway (36 %) [4] have shown that herbal remedies are commonly used to treat acute illnesses and pregnancy-related issues. In the Western world, pregnant women most frequently use herbs to treat conditions such as echinacea (common cold), cranberry (urinary tract irritation), ginger (nausea), and raspberry leaf (as a uterotonic) [5,6].

The prevalence of herbal medicine use varies from place to place. A systematic review study revealed that the worldwide prevalence of herbal use was 32.4 % [7]. The prevalence of use of herbal medicine during pregnancy in Saudi Arabia, and Palestine was 33 % and 40 % respectively [8,9]. In sub-Saharan Africa, the prevalence of herbal medicine use among pregnant women varies. The prevalence of use of herbal medicine in Uganda [10], Kenya [11], Sierra Leone [12], and eastern Kenya [13], was 20 %, 12 %, 62.7 %, and 100 % respectively.

There are regional variations in the prevalence of herbal use among pregnant women in Ethiopia. The prevalence of herbal medicine use was 22.6 % in Woldiya, Ethiopia [14], 36.3 % in Debre tabor, Ethiopia [15], 73.1 % in southern Ethiopia [16], 48.6 % in Gondar, Ethiopia [17], and 504.% in western Ethiopia [18]. According to a systematic review and meta-analysis study, the common herbal medicines used by pregnant women in Ethiopia were *ginger, damakasse, garlic, tenaadam, and eucalyptus* [19].

Evidence showed that the most common reasons for the use of herbal medicines were for the treatment of gastrointestinal disorders and common cold symptoms [20]. In addition to deep-rooted beliefs, customs, and rational attitudes, lack of knowledge and ignorance of the effects of practices help maintain the use of herbal medicines [21]. Several studies showed that educational status, occupational status, residency, history of herbal use, and maternal illness during pregnancy were significantly associated with the use of herbal medicine use among pregnant women [17,19,22].

In Ethiopia, certain practices involve the use of herbal medicine aimed at protecting mothers and newborns from perceived dangers and adverse effects [23,24]. However, these traditional embedded practices have an impact on the health of mothers and their newborns. Some evidence illustrated that the use of herbal medicines is not associated with adverse maternal and fetal outcomes [25] and others showed that herbal medicines are associated with adverse fetal outcomes such as abortion, congenital malformations, and fetal death [26]. This indicated that the association between the use of herbal medicines and adverse maternal outcomes is not well established and requires further investigation. Furthermore, the majority of studies in Ethiopia were conducted in health facilities that possibly excluded women who did not have antenatal care visits and there is limited research on these issues in community settings [15,18,22]. Therefore, the purpose of this study is to explore the association between the use of herbal medicines during pregnancy and adverse fetal outcomes among mothers in eastern Ethiopia.

## 2 Methods

### 2.1 Study setting and period

This study was carried out at the Health and Demographic Surveillance Sites (HDSS) in the eastern Ethiopian regions of Haramaya, Harar, and Kersa. The capital of the Harari Regional State is Harar, which contains 19 kebeles, or subdistricts. Twelve of these kebeles are part of the urban HDSS, which was started in 2011. Founded in 2007, Kersa HDSS is located in Kersa district in eastern Hararghe, Oromia region, Eastern Ethiopia. This site includes 24 representative kebeles (of 38 in the district) under health and demographic surveillance [27], but representativeness beyond the district level cannot be assumed. Four kebeles from Harar, Haramaya, and Kersa were used for this investigation. Basic sociodemographic information, Global Positioning System (GPS) locations, and comprehensive home characteristics are included in the database for each HDSS site. The Haramaya HDSS catchment area includes 12 of the 34 rural kebeles in Haramaya district. The total number of households in the HDSS was 17 461. The total baseline population was 99 898 (51 259 men and 48 639 women), of whom 23.86 % were women of reproductive age [28]. The study was carried out from February 2023 to November 2023.

### 2.2 Study design and population

A community-based cross-sectional study design was conducted among 882 randomly selected mothers. All selected mothers who gave birth in the past six months prior to the study and currently resided in Kersa, Haramaya, and Harar HDSS, eastern Ethiopia, were the study populations. All postnatal mothers who were unable to respond to the survey questionnaire during the data collection period due to severe illness, and unable to communicate verbally and mentally were excluded from the study.

### 2.3 Sampling size determination and sampling procedure

The sample size was calculated using a single population proportion formula with confidence level assumptions at 95 % = 1.96, a margin of error (d) = 0.03, and a reasonable proportion of herbal medicine use (P = 0.226) from a previous study conducted in Amhara region [14] and by adding 2 % design effect and a non-response rate of 5 %, the final sample size became 882.

A simple random sampling method was used to select mothers. There are 24 rural kebeles (Kersa and Haramaya) and 12 urban kebeles (Harar) in HDSS; two kebeles were chosen at random from each location. The distribution of the sample size among the six kebeles was based on the size of their respective populations. Lastly, using a simple random sampling method (lottery method) based on the sampling frame retrieved from HDSS (N = 8330) (N = 2,784, 1908 and 3621 for Kersa, Haramaya, and Harar respectively), study participants who were included in the study from each kebele were selected. A total of 295 samples were assigned to Kersa HDSS, 202 samples to Haramaya HDSS, and 384 samples to Harar HDSS.

### 2.4 Data collection methods and quality assurance

Data were collected using a structured questionnaire administered by interviewers, along with a review of records from the Health and Demographic Surveillance System (HDSS) sites in Kersa, Haramaya, and Harar. The questionnaire was adapted from established instruments with proven validity and reliability in similar populations [[16], [17], [18]]. The instrument was initially developed in English and subsequently translated into Amharic and Afaan Oromo to ensure accessibility for participants in the study area. To maintain accuracy and consistency, the translation process involved back-translation into English. The tool contains four sections such as; socio-demographic characteristics related to factors such as age, residency, educational status, marital status, and monthly income level; obstetric-related factors such as Antenatal care (ANC) follow-up visit, gravidity, complication during recent pregnancy, history of fetal loss, place of delivery and mode of delivery; herbal use such as type of use of herbal medicine, reason for use, route of administration, and side effects of herbal use.

To ensure validity, the questionnaires were pre-tested on 5 % of the sample of respondents before the actual data collection in Dre Dawa city. This pretest helped refine the questions for clarity, appropriateness, and cultural relevance, ensuring that the instrument accurately measured what it was intended to. To assess reliability, the structured questionnaire was administered consistently across all participants, and interviewers were trained to follow a standard protocol, minimizing interviewer bias and ensuring uniformity in the data collection process. Data were collected by well-trained 10 BSc midwives and supervised by 4 supervisors for data quality.

### 2.5 Operational definitions

**Herbal use:** is the use of parts of active ingredients of plants, other plant materials, or their combinations as part of traditional or conventional medicine and are not fully integrated into the dominant health care system [29].

**Adverse fetal outcomes**: The presence of at least one of the following (stillbirth, low birth weight, preterm, and congenital anomaly) [30].

### 2.6 Statistical analysis

The data were exported to Stata version 18 for analysis. Data were compiled using a descriptive statistical analysis. Frequencies, tables, and figures were used in the data presentation.

To explore the relationship between the independent variables and the use of herbal medicine, we employed a Poisson regression analysis model with robust variance estimation. This generalized linear model is specifically designed to directly estimate prevalence ratios in cross-sectional studies. Notably, when applied to binomial data, the Poisson regression with robust variance avoids convergence issues. Furthermore, in cases where the outcome variable is common, particularly with a prevalence exceeding 10 %, the odds ratio may overestimate the prevalence ratio, making the Poisson model a more appropriate choice for our analysis. Variables with p-values less than 0.25 in the bivariable analysis were included in the multivariable analysis. To find out if there was multicollinearity between the independent variables, the variance inflation factor (VIFs) and the correlation matrix for the regression coefficients were utilized. The VIF values were all below 2, suggesting that multicollinearity is not a significant concern. Additionally, the matrix indicated that the correlations were generally low. The Bayesian information criterion and Akaike's criteria were used to choose the final model. Pearson's Chi-square and the Hosmer-Lemeshow goodness-of-fit test were used to evaluate the model's fitness. To report the data, adjusted prevalence ratios (APRs) with a 95 % confidence interval were used. A P value less than 0.05 was designated as the statistically significant level.

## 3 Result

### 3.1 Socio-demographic characteristics

Of the total of 882 sampled mothers, 866 mothers participated in the study with a response rate of 98.2 %. Of these, 596 (68.8 %), and 567 (65.47 %), of the mothers were under 35 years of age and came from urban areas respectively. About 754(87.1 %) of mothers were married. Regarding the educational status of mothers, 432 (49.88 %) of the mothers completed the secondary education level. Four hundred and sixty-one (53.2 %) mothers had a middle economic status. Almost three-fourths, 641 (74.02 %), of mothers have health insurance. Regarding the time to reach the hospital, 408 mothers responded that less than 30 min is required to reach the hospital (Table 1).

**Table 1:** Sociodemographic characteristics of mothers in eastern Ethiopia, Ethiopia, 2023 (n = 866).

Variable	Frequency	Percentage
Age mothers		
<35 years	596	68.8
≥35 years	270	31.2
Residency		
Urban	567	65.47
Rural	299	34.53
Educational status of mothers		
Illiterate	82	9.47
Primary education	102	11.78
Secondary education	432	49.88
Collage and above	250	28.87
Marital status		
Not married	112	12.9
Married	754	87.1
Income status		
Low	364	42.1
Medium	461	53.2
High	41	4.7
Occupational status of mothers		
Housewife	198	22.86
Merchant	212	24.48
Government/private employee	424	48.96
Farmer	32	3.7
Have health insurance		
Yes	641	74.02
No	225	25.98
Time to reach a health facility		
Below 30 min	408	47.11
30–60 min	298	34.41
Above 1 h	160	18.48

### 3.2 Obstetrics-related characteristics

Of the total of participants, 605 (69.86 %) of the mothers were multigravida. More than three-fourths (79.21 %) of the mothers had ANC follow-up. There were 86 (9.93 %) mothers with a history of neonatal loss. More than a third (27.02 %) of mothers experienced obstetric complications in the last pregnancy. There were 800 (92.38 %) mothers with a single pregnancy. There were 41 (4.73 %) home deliveries. Of the total of the respondents, 61 (7.04 %) of the mothers experienced adverse fetal outcomes in recent pregnancy (Table 2).

**Table 2:** Obstetrics-related characteristics of the mothers in eastern Ethiopia, 2023 (n = 866).

Variables	Frequency	Percentage
ANC follow-up		
Yes	686	79.21
No	180	20.79
Gravidity		
Primigravida	261	30.14
Multigravida	605	69.86
History of neonatal loss		
Yes	86	9.93
No	780	90.07
Presence of pregnancy complications		
Yes	234	27.02
No	632	72.98
No fetus in the last pregnancy		
Single	800	92.38
Multiple	66	7.62
Place of delivery		
Home	41	4.73
Health facility	825	95.27
Experience adverse fetal outcome in recent pregnancy		
No	805	92.96
Yes	61	7.04

ANC = Antenatal Care.

### 3.3 Herbal medicine use

The prevalence of the use of herbal medicine among pregnant women in eastern Ethiopia was 43.2 % (95 % CI 39.9,46.5). The most commonly used herbal medicine were *Ocimum lamiifolium* 109 (12.6 %), *Pumpkin fruit* 68(7.9 %), *Rutachalepensis* (tenadam) 61(7 %), *Feto* 45(5.2 %), and *Ginger* 41(4.7 %). The most common indications for the use of herbal medicines were Vomiting 76(20.3 %), Fever 76(20.3 %), Taeniases 64(17.1 %), Nausea 54(14.4 %), and Abdominal pain 36 (9.6 %). Of the mothers who used herbal medicine in the recent pregnancy, 79 (21.1 %) experienced unwanted side effects. The most common reason for the use of herbal Medicine was maternal illness during pregnancy 151 (40.4 %) and perceiving its safety during pregnancy147 (39.3 %) (Table 3).

**Table 3:** Herbal medicine use and related characteristics of mothers in eastern Ethiopia, Ethiopia, 2023 (n = 866).

Variable	Frequency	Percentage
Herbal Medicine Uses during Recent Pregnancy		
Yes	374	43.19
No	492	56.81
Type of Herbal Medicine Used by mothers (n = 374)		
*Zingiber officinale* (*Ginger)*	41	10.9
*Allium sativum (Garlic)*	4	1.1
*Eucalyptus* spp. *(Eucalyptus)*	26	6.9
*Leonotis nepetifolia (Tenaadam)*	61	16.3
*Ocimum lamiifolium (Damakese)*	109	29.4
*Ficus thonningii (Feto)*	45	12
*Cucurbita pepo (Pumpkin fruit orDuba fire)*	68	18.2
*Linum usitatissimum (Telba)*	20	5.3
Indication for Herbal Medicine Use (n = 374)		
Nausea	54	14.4
Vomiting	76	20.3
Abdominal pain	36	9.6
Cold	15	4
Amebiasis	11	2.94
Fever	76	20.3
Taeniases	64	17.1
Typhoid fever	1	0.27
Tonsilitis	32	8.5
Diarrhea	9	2.4
Route of medicine administration (n = 374)		
Oral	315	84.2
Topical	40	10.7
Intra vaginal	1	0.3
Intra nasal	18	4.8
Gestational age where medication administered (n = 374)		
At specific gestation	137	36.7
Throughout pregnancy	237	63.3
Experience unwanted side effects of medicine (n = 374)		
Yes	79	21.1
No	295	78.9
Common reported side effects (n = 79)		
Burning sensation	51	64.6
Vomiting	17	21.5
Diarrhea	5	6.3
Abdominal pain	6	7.6
Reason for herbal use (n = 374)		
Safe in pregnancy	147	39.3
Believes in the effectiveness of herbal medicines	5	1.3
Part of our culture to use it	71	19
Maternal illness during pregnancy	151	40.4
Source of information for herbal use (n = 374)		
Traditional medicine healers	72	19.3
Self-preparation	25	6.7
Family members	273	73
Media	4	1.07

### 3.4 Distribution of herbal medicine use among different type of adverse fetal outcomes

*Leonotis nepetifolia (Tenaadam)* use was high among mothers who experienced stillbirth compared to other adverse fetal outcomes. *Ocimum lamiifolium (Damakese)* use was high among mothers who experienced preterm birth compared to other adverse fetal outcomes (Table 4).

**Table 4:** Distribution of herbal medicine use among different types of adverse fetal outcomes in eastern Ethiopia, 2023.

Herbal Medicines	Stillbirth		LBW		Preterm		Congenital anomaly	
	Yes	No	Yes	No	Yes	No	Yes	No
*Ginger*	6	35	5	36	2	39	0	41
*Garlic*	0	4	0	4	0	4	0	4
*Eucalyptus*	5	21	0	26	3	23	0	26
*Tenaadam*	14	47	1	46	0	61	3	58
*Ocimum lamiifolium (Damakese)*	9	100	5	104	11	98	5	104
*Feto*	4	41	1	44	3	42	6	39
*Pumpkin fruit (Duba fire)*	6	62	0	68	5	63	5	63
*Linen (Telba)*	0	20	2	18	0	20	0	20

LBW = Low Birth Weight.

### 3.5 Factors associated with the use of herbal medicine

Rural residency, history of neonatal loss, presence of complications of obstetrics during recent pregnancy, lack of ANC follow-up, multigravida, and adverse fetal outcomes were significantly associated with the use of herbal medicines in the bivariate Poisson regression model. After controlling for possible confounders in the multivariate Poisson regression model, variables such as rural residency, presence of obstetric complications during recent pregnancy, and having adverse fetal outcomes were significantly associated with the use of herbal medicines.

The prevalence of herbal medicine use among rural residents was 1.27 times [APR = 1.27, 95%CI (1.01,1.59)] higher than urban residents. The prevalence of herbal medicine use among mothers who had obstetric complications in recent pregnancy increased by 42 % compared to mothers without the condition [APR = 1.42, 95%CI: (1.14,1.78)]. The prevalence of herbal use among mothers who experienced adverse fetal outcomes was 1.59 times that of mothers who did not have the conditions [APR = 1.59, 95%CI: (1.05,2.2)] Table 5.

**Table 5:** Factors associated with herbal Medicine Use among pregnant women in eastern Ethiopia, Ethiopia, 2023.

Variables		Herbal Use		CPR (95 % CI)	APR (95%CI)
		No	Yes		
Residency	Urban	353	214	1	1
	Rural	139	160	1.42(1.16,1.74)^b^	1.27(1.01,1.59)^a^
Age	<35 years	352	244		
	≥35 years	140	130	1.18(0.95,1.45)	0.98(0.77,1.25)
History of neonatal loss	Yes	29	57	1.63(1.23,2.16) ∗∗	1.14(0.82,1.61)
	No	463	317	1	1
ANC follow-up	Yes	405	281	1	1
	No	87	93	1.26(1,1.59)^a^	0.93(0.69,1.25)
Presence of complications during pregnancy	Yes	98	136	1.54(1.25,1.91)^b^	1.42(1.14,1.78)^b^
	No	394	238	1	1
Place of delivery	Health facility	476	349	1	1
	Home	16	25	1.44 (0.96,2.16)	1.22(0.77,1.91)
Gravidity	Primigravida	166	95	1	1
	Multigravida	326	279	1.27(1.01,1.60)^a^	1.12(0.86,1.45)
Experience of adverse fetal outcomes	No	478	327	1	1
	Yes	14	47	1.90(1.40,2.58)^b^	1.52(1.05,2.2)^b^

a Significant with P < 0.05 and.

b Significant with P < 0.001, CI= Confidence Interval, CPR= Crude Prevalence Ratio, APR = Adjusted Prevalence ratio.

## 4 Discussion

This study found that 43.2 % of pregnant women in Eastern Ethiopia used herbal medicines, with the most commonly used being *Ocimum lamiifolium* (29.1 %), *pumpkin fruit* (18.2 %), and *Rutachaleensis* (16.3 %). The use of herbal medicines was significantly associated with factors such as rural residency, obstetric complications in recent pregnancies, and adverse fetal outcomes. The study found that the prevalence of herbal medicine use among pregnant women in eastern Ethiopia was found to be 43.2 % (95 % CI:39.9,46.5). The finding of this study is in harmony with a study done in Palestine (40 %) [9]. The possible justification may be the similarity in socioeconomic status of the participants.

The finding of this study is higher than studies done in Woldiya, Ethiopia 22.6 % [14], Debre tabor, Ethiopia (36.3 %) [15], Uganda (20 %) [10], Kenya (12 %) [11], and Saudi Arabia (33 %) [8]. This finding is also lower than studies done in southern Ethiopia (73.1 %) [16], Gondar, Ethiopia (48.6 %) [17], western Ethiopia (50.4 %) [18], Dessie, Ethiopia (51.2 %) [22], and Sierra Leone (62.7 %) [12]. A possible justification could be the difference in sample sizes; the sample size of the current study is larger than studies conducted in Ethiopia and other African countries,. The other potential discrepancy is the study setting; the studies were conducted in health institutions. The current study is conducted in a community setting, which can include women who miss ANC follow-up. A systematic review and meta-analysis conducted in Ethiopia highlighted that the effects of available herbal medications on the fetus have not been thoroughly studied, making it difficult to rule out potential adverse maternal and fetal outcomes. Therefore, traditional healers and medical professionals need to collaborate in disseminating critical information about these medications to mitigate any negative effects [19]. To address safety concerns and negative effects, more research is necessary, as this study suggests that herbal medications are commonly used in eastern Ethiopia. Additionally, healthcare professionals should be educated about the potential risks associated with herbal medicine use among pregnant women. In this study, rural residency was significantly associated with the use of herbal medicines. The prevalence of herbal medicine use among rural residents was 1.27 times [APR = 1.27, 95%CI (1.01,1.59)] higher than urban residents. This finding is consistent with previous studies conducted in Ethiopia and Nigeria [14,17,18,31]. This might be due to mothers living in rural areas being more prone to experience problems with the accessibility and affordability of contemporary medicine, leading them to turn to herbal remedies. The high prevalence of herbal medicines use among rural residents may also be due to the lack of information available on the risks associated with the use of herbal medicines, as well as the fact that pregnant women in rural areas have less access to health facilities than those in urban areas [18].

We found that experiencing obstetric complications during a recent pregnancy was significantly associated with the use of herbal medicines. The prevalence of herbal medicine uses among mothers who had obstetric complications in recent pregnancy was 1.42 times higher compared to mothers without the condition. This finding is in line with studies done in Ethiopia [15,19]. This may be due to the fact that most women in this study accessed herbalist information and may be convinced to use medicines to treat the complications. The other possible reason may be the previous experience of using medication to treat health problems [10,19]. Furthermore, in this study, nearly half of mothers who used herbal medicines believed that herbal medicines are safe to use during pregnancy.

In this study, the experience of adverse fetal outcomes in recent pregnancy was significantly associated with the use of herbal medicines. The prevalence of herbal medicine uses among mothers who experienced adverse fetal outcomes was 1.59 times that of mothers who did not have the condition [APR = 1.59, 95%CI: (1.05,2.2)]. This finding is in agreement with studies done in China [32] and Malaysia [33]. However, the finding is in contrast with a study done in Sweden [25]. This may be attributed to prior experience with using medications to address health issues. Consequently, it is crucial to evaluate both the risks and benefits of herbal medicines by conducting further safety investigations and clinical trials with larger sample sizes to confirm their safety during pregnancy [10,19].

### 4.1 Clinical implications

The clinical implications of this study are significant and multifaceted, particularly in the context of complementary and alternative medicine (CAM), including herbal products. With the increasing global interest in CAM, healthcare providers need to recognize both the potential benefits and risks associated with herbal medicine use [[34], [35], [36], [37]]. Herbal medicines, often perceived as natural and safe, are widely used by pregnant women, but their effects on maternal and fetal health are not always well understood. This study highlights the importance of incorporating herbal medicine use into routine prenatal care assessments. Healthcare providers should provide appropriate counseling to pregnant women about the potential risks and benefits of herbal products, ensuring that they are informed and supported in their choices. Pregnant women who report using herbal medicines should undergo a thorough risk assessment and be monitored throughout their pregnancy. The findings of this study can help inform the development of evidence-based guidelines or protocols tailored to the local context, which take into account cultural practices and beliefs surrounding herbal medicine use. Overall, this study underscores the growing relevance of integrating knowledge about herbal medicine use into contemporary healthcare practices. As the use of CAM continues to rise, it is crucial to develop comprehensive strategies that promote informed decision-making and ensure the safety and well-being of both pregnant women and their unborn babies in Eastern Ethiopia.

### 4.2 Research implications

The study highlights the need for further research to better understand the mechanisms underlying the association between herbal medicine use during pregnancy and adverse fetal outcomes. Collaborative efforts between healthcare providers, researchers, traditional healers, and community members can help identify effective interventions and strategies to mitigate risks and improve maternal and child health outcomes in Eastern Ethiopia.

### 4.3 Strengths and limitations

The strength of the study is that study was conducted in multiple areas of eastern Ethiopia. The limitation of this study was that it might not indicate a cause-effect relationship due to the nature of the study. There may be recall bias in the listing of the herbs. Furthermore, the study was conducted in eastern Ethiopia, which may limit the generalizability of the findings to other areas with different demographic, cultural, or healthcare contexts.

## 5 Conclusion

The study found that more than two out of five mothers used herbal medicines during their recent pregnancies. It identified significant associations between herbal medicine use and factors such as rural residency, obstetric complications during the current pregnancy, and adverse fetal outcomes. Therefore, it is crucial to verify the efficacy and safety of herbal medicines before they are used during pregnancy. A collaborative strategy involving herbalists, communities with strong beliefs in herbal remedies, and healthcare professionals should be developed to mitigate the risks associated with indiscriminate herbal medicine use and to address the diverse health needs of pregnant women.

## CRediT authorship contribution statement

**Tamirat Getachew:** Writing – review & editing, Writing – original draft, Visualization, Validation, Supervision, Investigation, Formal analysis, Conceptualization. **Abera Kenay Tura:** Writing – review & editing, Writing – original draft, Visualization, Validation, Supervision, Methodology, Conceptualization. **Merga Dheresa:** Writing – review & editing, Writing – original draft, Visualization, Validation, Software, Methodology, Formal analysis, Data curation. **Shamble Niguse:** Writing – review & editing, Writing – original draft, Visualization, Validation, Investigation, Funding acquisition, Conceptualization. **Bikila Balis:** Writing – review & editing, Writing – original draft, Visualization, Project administration, Methodology, Funding acquisition, Formal analysis. **Lemma Demissie Regassa:** Writing – review & editing, Writing – original draft, Supervision, Resources, Methodology, Investigation, Formal analysis. **Addis Eyeberu:** Writing – review & editing, Writing – original draft, Visualization, Validation, Supervision, Methodology, Investigation, Formal analysis, Conceptualization.

## Ethics approval and consent to participate

Ethical clearance was obtained from Haramaya University, the Faculty of Health and Medical Sciences, Institutional Health Research Ethics Review Committee (IHRERC). The ethical approval ref.no was IHRERC/225/2021. Informed, voluntary, written, and signed consent was obtained from study participants before data collection. The study was carried out in accordance with the Declaration of Helsinki.

## Consent to publication

Non-applicable.

## Availability of data and materials

All necessary data are included in the manuscript. However, additional data are available from the corresponding author on reasonable request.

## Funding

This work was supported by 10.13039/501100004845Haramaya University [grant number HURG_2021_02_04_71].

## Declaration of competing interest

The authors declare that they have no known competing financial interests or personal relationships that could have appeared to influence the work reported in this paper.
