# Supplementary material for: Association between the use of herbal medicines during pregnancy and adverse fetal outcomes among mothers in eastern Ethiopia, 2023
Source: Heliyon. 2024 Dec 6;10(24):e40941. doi: 10.1016/j.heliyon.2024.e40941 (PMC11681852; doi:10.1016/j.heliyon.2024.e40941)
Supplement: Multimedia component 1 [file mmc1.docx]

**Questionnaire (English Version)**

Questionnaire ID no.________

**General instructions**

1.Choose one of the alternatives given for each question

2.If your answer is not found among the given alternative tell your answer to the interviewer

|  | **Part I Socio-demographic characteristics of the respondent** | | | |
| --- | --- | --- | --- | --- |
| **S/No** | **Questions** | Response | Code | Skip to |
| 101 | Age of respondent (in years) | _____ |  |  |
| 102 | Residence | 1. 1. Urban 2. 2. Rural |  |  |
| 103 | Current marital status | 1. 1. Single 2. 2. Married 3. 3. Divorced   4. Widowed |  |  |
| 104 | Educational status of the mother | 1. 1. No education 2. 2. Primary 3. 3. Secondary   4. Collage and above |  |  |
| 105 | Educational status of the husband | 1. No education  2. Primary  3. Secondary  4. Collage and above |  |  |
| 106 | Occupational status of the mother | 1. 1. Housewife 2. 2. Merchant 3. 3. Government employee 4. 4. Private employee 5. 5. Farmer 6. 6. Others |  |  |
| 107 | Occupational status of the husband | 1. Housewife  2. Merchant  3. Government employee  4. Private employee  5. Farmer  6. Others |  |  |
| 108 | Religion | 1. 1. Orthodox 2. 2. Muslim 3. 3. Protestant 4. 4. Catholic   5. Others (Specify)___ |  |  |
| 109 | Monthly income of the house hold in birr | …………………ETB |  |  |
| 110 | Number of family living together | **------------------** |  |  |
| 111 | Time to reach health facility (Hr./foot) | **-------------------**Hrs./foot |  |  |
| 112 | Do you have health insurance? | 1. Yes 2. No |  |  |
|  | **Part II obstetrics characteristics of the respondent** | | | |
| 201 | Number of pregnancy(gravidity) | _____________ |  |  |
| 202 | Number of childbirth(parity) | _____________ |  |  |
| 203 | Birth interval (current) | _____________ |  |  |
| 204 | History of neonatal loss | 1. 1. Yes   2. No |  | 206 |
| 205 | Number of neonatal losses | ________ |  |  |
| 206 | ANC follow-up | 1. 1. 2Yes 2. 2. No |  |  |
| 207 | No of visit | 1. _____ |  |  |
| 208 | Presence of Complication during pregnancy | 1. 1. Yes   2. No |  | 215 |
| Type maternal of complications  Did the mother developed | | |  |  |
| 209 | APH (antepartum hemorrhage) | 1. 1. Yes   2. No |  |  |
| 210 | Pregnancy-induced hypertension | 1. 1.Yes   2. No |  |  |
| 211 | Preterm labor | 1. 1. Yes   2. No |  |  |
| 212 | Premature rupture of membrane | 1. 1. Yes   2. No |  |  |
| 213 | Others specify | _________ |  |  |
| 214 | No. of fetus in this/last pregnancy | 1. 1. Single   2. Multiple |  |  |
| 215 | Onset of labor | 1. 1. Spontaneous 2. 2.  Induced 3. 3. C/S before the onset 4. 4. Other _____ |  |  |
| 216 | Duration of labor | ________hrs. |  |  |
| 217 | Complications during labor? | 1. 1. Yes   2. No |  | 412 |
| 218 | What was your response to these obstetrics complications? | 1. Go to health facility 2. Consulted traditional healers 3. Consulted religious leaders 4. Taking home remedy 5. Others (specify)____ |  |  |
| **Type of labor complications** | | |  |  |
| 219 | Obstructed labor | 1. 1. Yes   2.No |  |  |
| 220 | Prolonged labor | 1. 1. Yes   2. No |  |  |
| 221 | PPH (massive postpartum hemmorhage) | 1. 1. Yes   2. No |  |  |
| 222 | Sepsis | 1. 1. 2Yes   2. No |  |  |
| 223 | Eclampsia (loss of consiousness due to hypertansion) | 1. 1. Yes   2. No |  |  |
| 224 | Other specify | _______ |  |  |
| 225 | Mode of delivery | 1. 1. SVD (spontaneous vaginal delivery   2. C/S (caesarean section)  3. Vacuum  4. Forceps  5. Other specify___ |  |  |
| 226 | Birth attendant | 1. 1. TBA (traditional birth attendant)   2. Relatives/families  3. Health professionals  4. Other specify ____ |  |  |
| 227 | Previous bad obstetrics history | 1. 1. Yes 2. 2. No |  |  |
| 228 | Bad obstetrics history/ specify | 1. Abortion, 2. stillbirth, 3. neonatal loss 4. other specify___ |  |  |
|  |  |  |  |  |
|  | **Part III herbal use during pregnancy and child birth** | | | |
| 301 | Have you use herbals during current pregnancy/child birth | 1. Yes  2. No |  |  |
| 302 | If yes which one | 1. ginger 2. garlic (Allium sativum) 3. eucalyptus 4. tenaadam 5. damakesse (Ocimum lamiifolium) 6. feto 7. omore 8. anamura 9. barewa 10. duba fire 11. limich 12. tosign 13. koso 14. Telba 15. others(specify)---------------------- |  |  |
| 303 | Indications for using the herbal medicine during pregnancy | 1. nausea 2. vomiting 3. abdominal pain 4. cold 5. malaria 6. amoebiasis 7. fever, 8. Taeniasis 9. typhoid fever 10. tonsillitis, 11. Diarrhea 12. Others(specify)-------------- |  |  |
| 304 | Routes through which herbal medicines were used | 1. Oral 2. Topical 3. Intra vaginal 4. Intranasal 5. Other (specify)_____ |  |  |
| 305 | The gestational age at which you used the herbal medicine | 1. _____weeks 2. Throughout pregnancy |  |  |
| 306 | Experienced untoward effect on post administration | 1. Yes 2. No |  |  |
| 307 | What untoward effects on post administration | 1. Burning sensation 2. Vomiting 3. Dizziness 4. Malaise 5. Headache 6. Diarrhoea 7. Abdominal pain 8. Rashes 9. Others(specify)------------- |  |  |
| 308 | Source of information for use of herbals | 1. Traditional medicine healers 2. Self-preparation 3. Family members 4. Media 5. Others(specify)-------------- |  |  |
| 309 | Reasons to use herbal medicine over other medicine | 1. Safe in pregnancy 2. Believes in effectiveness of herbal medicines 3. is part of our culture to use it 4. is always available when I need them 5. To prevent miscarriages/abortion 6. Maternal illness during pregnancy 7. Others (specify)------------ |  |  |
